# Genome-wide association study unveils ascorbate regulation by PAS/LOV PROTEIN during high light acclimation

**DOI:** 10.1093/plphys/kiad323

**Published:** 2023-06-02

**Authors:** Fayezeh Aarabi, Andrea Ghigi, Micha Wijesingha Ahchige, Mustafa Bulut, Peter Geigenberger, H Ekkehard Neuhaus, Arun Sampathkumar, Saleh Alseekh, Alisdair R Fernie

**Affiliations:** Central Metabolism, Max-Planck-Institute of Molecular Plant Physiology, Am Mühlenberg 1, Potsdam-Golm 14476, Germany; Central Metabolism, Max-Planck-Institute of Molecular Plant Physiology, Am Mühlenberg 1, Potsdam-Golm 14476, Germany; Central Metabolism, Max-Planck-Institute of Molecular Plant Physiology, Am Mühlenberg 1, Potsdam-Golm 14476, Germany; Central Metabolism, Max-Planck-Institute of Molecular Plant Physiology, Am Mühlenberg 1, Potsdam-Golm 14476, Germany; Department Biology I, Ludwig-Maximilians-University Munich, Planegg-Martinsried 82152, Germany; Plant Physiology, University of Kaiserslautern, Kaiserslautern D-67653, Germany; Central Metabolism, Max-Planck-Institute of Molecular Plant Physiology, Am Mühlenberg 1, Potsdam-Golm 14476, Germany; Central Metabolism, Max-Planck-Institute of Molecular Plant Physiology, Am Mühlenberg 1, Potsdam-Golm 14476, Germany; Crop Quantitative Genetics, Centre of Plant Systems Biology and Biotechnology, Plovdiv 4000, Bulgaria; Central Metabolism, Max-Planck-Institute of Molecular Plant Physiology, Am Mühlenberg 1, Potsdam-Golm 14476, Germany; Crop Quantitative Genetics, Centre of Plant Systems Biology and Biotechnology, Plovdiv 4000, Bulgaria

## Abstract

Varying light conditions elicit metabolic responses as part of acclimation with changes in ascorbate levels being an important component. Here, we adopted a genome-wide association-based approach to characterize the response in ascorbate levels on high light (HL) acclimation in a panel of 315 Arabidopsis (*Arabidopsis thaliana*) accessions. These studies revealed statistically significant SNPs for total and reduced ascorbate under HL conditions at a locus in chromosome 2. Ascorbate levels under HL and the region upstream and within *PAS/LOV PROTEIN* (*PLP*) were strongly associated. Intriguingly, subcellular localization analyses revealed that the PLPA and PLPB splice variants co-localized with VITAMIN C DEFECTIVE2 (VTC2) and VTC5 in both the cytosol and nucleus. Yeast 2-hybrid and bimolecular fluorescence complementation analyses revealed that PLPA and PLPB interact with VTC2 and that blue light diminishes this interaction. Furthermore, PLPB knockout mutants were characterized by 1.5- to 1.7-fold elevations in their ascorbate levels, whereas knockout mutants of the *cry2* cryptochromes displayed 1.2- to 1.3-fold elevations compared to WT. Our results collectively indicate that PLP plays a critical role in the elevation of ascorbate levels, which is a signature response of HL acclimation. The results strongly suggest that this is achieved via the release of the inhibitory effect of PLP on VTC2 upon blue light illumination, as the VTC2-PLPB interaction is stronger under darkness. The conditional importance of the cryptochrome receptors under different environmental conditions suggests a complex hierarchy underpinning the environmental control of ascorbate levels. However, the data we present here clearly demonstrate that PLP dominates during HL acclimation.

## Teaser

PAS/LOV PROTEIN is a negative regulator of ascorbate in the dark by direct binding to VITAMIN C DEFECTIVE2, the key enzyme of the pathway.

## Introduction

Ascorbate (vitamin C), is a vital and ubiquitous antioxidant in all plant cellular compartments, being critical in scavenging the reactive oxygen species (ROS) in response to various stresses (Rosado-Souza et al. 2020). Besides its profound antioxidant function, it is a cofactor for multiple enzymes, including violaxanthin de-epoxidase, an enzyme involved in non-photochemical quenching (Smirnoff 2018), and 2-oxoglutarate-dependent dioxygenases (2-ODDs), including the 10 to 11 translocation enzyme that promotes DNA demethylation in human and algal cells (Blaschke et al. 2013; Xue et al. 2019). Moreover, several reports have demonstrated ascorbate acts as a chaperone for plant 2-ODDs functioning in hormone metabolism and synthesis of secondary metabolites including anthocyanins and glucosinolates (Tóth 2023). Several lines of evidence reported the importance of ascorbate in photosynthesis as it can regulate the chloroplastic electron transport chain by acting as an electron donor or acceptor for PSII (Mano et al. 2004; Nunes-Nesi et al. 2005; Tóth et al. 2009, 2011). Ascorbate is predominantly synthesized through the D-mannose/L-galactose (Smirnoff–Wheeler) pathway, being tightly regulated by the *VITAMIN C DEFECTIVE2* (*VTC2*) and *VTC5* paralogs encoding GDP-L-galactose phosphorylase (GGP), the key enzyme of the pathway (Bulley and Laing 2016; Fenech et al. 2021). Indeed, *vtc2vtc5* double mutants were utterly devoid of ascorbate, demonstrating the pivotal role of these genes in ascorbate biosynthesis (Dowdle et al. 2007). The transcription of *VTC2* and the activity of the GGP enzyme is highly responsive to environmental conditions, such as oxidative stress (Urzica et al. 2012; Vidal-Meireles et al. 2017) and exceptionally high light (HL) (Dowdle et al. 2007; Yabuta et al. 2007; Müller-Moulé 2008; Page et al. 2012; Schmitz et al. 2014; Laing et al. 2017). In line with increasing the expression of the *VTC2*, the size of the ascorbate pool also increases in plants under intense light, depending on the length of light exposure. *VTC2* and *VTC5* were identified to be induced in concert upon 24 h exposure to HL, leading to a 20-fold increase in the activity of the corresponding enzyme, GGP, and an increase in ascorbate levels (Dowdle et al. 2007). Ascorbate levels almost doubled after 3 d of continuous light in Arabidopsis leaves (Yabuta et al. 2007). Furthermore, relationships between light and respiration have been observed for ascorbate accumulation given that light is considered a major driving force of ascorbate accumulation in the light and disappearance in the dark (Bartoli et al. 2006). Besides GGP, L-galactono-1,4-lactone dehydrogenase (GLDH), the last enzyme of the pathway, demonstrated light regulation of ascorbate biosynthesis at the level of the enzyme activity (Smirnoff 2000; Bartoli et al. 2006; Fenech et al. 2019). Supplementing Arabidopsis plants with L-galactone-1,4-lactone (L-GalL; the precursor of ascorbate) under HL led to an upregulation of ascorbate levels and twice as high GLDH activities with higher respiration rates compared to the low-light grown plants (Bartoli et al. 2006). Moreover, redox control has been described as regulating Arabidopsis GLDH via reversible oxidation of a cysteine residue (Cys-340) leading to the inactivation of GLDH (Leferink et al. 2009). Such redox modification may be a route for light-dark regulation of this enzyme (Maruta 2022). Furthermore, a diurnal pattern for ascorbate increase in the light and decrease in the dark has been reported (Dutilleul et al. 2003; Tamaoki et al. 2003), mainly through the expression of *VTC2* rather than *GLDH* (Bartoli et al. 2006; Dowdle et al. 2007).

Despite the importance of VTC2 in ascorbate biosynthesis and its regulatory role in response to light, we need to learn more about the regulation of this enzyme. While it is known that a conserved upstream open reading frame (uORF) in the 5′-UTR of *VTC2* represses GGP translation in a feedback regulation manner (Laing et al. 2015), the uORF functionality in the light requires further investigation. In some attempts to remove the uORF region by genome editing resulted in higher ascorbate concentration in Arabidopsis, tomato, and lettuce (Laing et al. 2015; Zhang et al. 2018; Li et al. 2018a). In addition, a mutation in uORF of *VTC2* has been identified in an ethyl methanesulfonate (EMS)-mutagenized MicroTom population being responsible for a remarkable increase of ascorbate concentration in tomato fruits and impairment in flower and pollen development, suggesting the regulatory role of uORF-*VTC2* in redox regulation of plant (Deslous et al. 2021). The ascorbate-enriched mutant was further validated using CRISPR strategy by generating a tomato uORF-VTC2 mutant (Deslous et al. 2021).

Furthermore, a light-regulated *cis-*element located at −40 to −70 bp of its promoter has been reported to be crucial for the light regulation of VTC2 (Gao et al. 2011). Nevertheless, some positive and negative regulators for ascorbate biosynthesis have been identified (Rosado-Souza et al. 2020). The ethylene response factor98 (AtERF98) binds to the promoter of *VTC1*, encoding GMP, and enhances the expression of multiple pathway genes, including *VTC1*, *VTC2*, *GDH*, and *GLDH* (Zhang et al. 2012). However, to date, the role of AtERF98 is mainly studied under salt stress with no information available concerning the response to HL stress.

As mentioned above, light is a driving force for ascorbate biosynthesis; therefore, under continuous darkness, ascorbate levels decrease markedly (Truffault et al. 2017; Maruta 2022). In contrast to AtERF98, a negative regulator of ascorbate pathway genes, with the highest effect on *GME* and *VTC2* has been identified and named ascorbic acid mannose pathway regulator 1 (AMR1) (Zhang et al. 2009). *AMR1* expression decreases at a HL intensity when ascorbate increases (Zhang et al. 2009). Therefore, AMR1 appears to have a regulatory function in the light-dark regulation of ascorbate levels. Another component of light-dark regulation of ascorbate levels is a subunit of the photomorphogenic factor, COP9 signalosome, Constitutive photomorphogenic9-signalosome subunit 5B (CSN5B), which interacts with GDP-mannose pyrophosphorylase (VTC1) in the dark and thereby promotes its degradation through the 26S proteasome pathway, resulting in lower ascorbate content (Wang et al. 2013).

Over the last few years, metabolic genome-wide association studies have been used for various species to identify genetic determinants of plant metabolism, providing a better understanding of metabolic diversity along the way (Fang and Luo 2019). For example, a GWAS study on 302 tomato accessions identified a positive regulator for ascorbate content in tomato fruits from the family of basic helix–loop–helix (bHLH) transcription factors SlbHLH59 (Ye et al. 2019). SlbHLH59 directly binds to the promoter of key genes of the ascorbate pathway and upregulates ascorbate levels (Ye et al. 2019). A few GWAS studies have been performed to study light acclimation responses in Arabidopsis. For example, van Rooijen et al. (2017) performed a GWAS study to identify quantitative trait loci involved in the Arabidopsis photosynthetic acclimation response (van Rooijen et al. 2017). As a result, they discovered *YELLOW SEEDLING1 (YS1)YS1*, a determinant of RNA editing of plastid-encoded genes (anterograde signaling; van Rooijen et al. 2017). However, GWAS studies under stress remain relatively rare, to date, largely focusing on either extended darkness or water-deficit stress (Zhang et al. 2021; Zhu et al. 2022b), despite the fact that convincing arguments have been made that GWAS studies are superior is also carried out under light stress conditions (Zhu et al. 2022a). In this study, we adopted a GWAS approach to identify novel regulatory mechanisms of ascorbate signaling during HL acclimation by using around 300 different accessions of *Arabidopsis thaliana*. As a result, we obtained statistically significant SNPs for total and reduced ascorbate under high-light conditions found in a locus in chromosome 2. Leading SNPs identified AT2G02710, encoding the PAS/LOV PROTEIN (PLP), a putative blue light receptor (Ogura et al. 2008b). Because it has been shown that PLP interacts with VTC2 (Ogura et al. 2008b), and since the putative photoreceptive function of PLP was coherent with a role in ascorbate regulation in HL, this gene, among other genes of the locus, captured our attention.

PLP was proposed as blue light receptors in plants due to the existence of an N-terminal Per-ARNT-Sim (PAS) and a C-terminal light-oxygen-voltage (LOV) domain in its structure, which form a distinct group in the analyzed phylogenetic trees of LOV domain proteins (Ogura et al. 2008b; Ishikawa et al. 2009; Kasahara et al. 2010). After identifying 2 tandem PAS-like domains in plant phototropins, PHOT1, and PHOT2, the term LOV domain was introduced to distinguish them from other PAS domains (Crosson et al. 2003; Möglich et al. 2009). In other words, the LOV protein domains form a subset of the diverse PAS domain superfamily, which has distinct roles in cellular signaling processes using flavin chromophore as a module for sensing blue light in plants and fungi (Christie et al. 1999; Crosson et al. 2003; Ogura et al. 2008a). LOV2 domain of phototropins contains a conserved cysteine residue (S-4a-FMN cysteine), that upon blue light illumination, binds to 2 Flavin MonoNucleotide (FMN) chromophores and reverses under dark conditions (Paik and Huq 2019). Similar to the phototropins, the PLP protein has such conserved cysteine residue (UniProt; Ogura et al. 2008a); therefore, similar conformational switches under dark-light conditions are conceivable for PLP in Arabidopsis. Furthermore, Kasahara et al. (2010) revealed that tomato PLP binds FMN in both PAS and LOV domains. However, the PAS domain of the PLP lacks the key cysteine residue for light perception (Ogura et al. 2008a). Unlike phototropins whose functional roles have been deciphered in multiple signaling processes, such as blue light-mediated signaling of plant tropism, chloroplast movement, and stomatal opening (Kinoshita et al. 2001; Pedmale and Liscum 2007; Demarsy et al. 2012; Kong and Wada 2016), the molecular function of PLP proteins remained elusive. Ogura et al. (2008b) using a nontargeted Y2H, identified positive interactions between VTC2 and PLP proteins (PLPA and PLPB isoforms, encoded by different splice variants), demonstrating diminished interaction under blue light. In line with the former study, we confirmed VTC2-PLPA and VTC2-PLPB interactions in both yeast and plants. We also observed that VTC2 forms much stronger interactions with PLPB and PLPA under dark than blue light conditions, confirming the blue light responsiveness of these proteins. To clarify the function of PLP in light-dark regulation of ascorbate and further verify the role of the GWAS candidate gene in ascorbate regulation, we isolated the *plp* knockout mutants, which demonstrated higher ascorbate levels in all light conditions compared to wild-types (WTs), indicative of responsiveness of this protein to other light spectra in addition to blue light. That being said, our results demonstrate that PLP could have a negative effect on VTC2 in the dark by direct protein binding; therefore, ascorbate is strongly reduced, whereas this interaction is released under the HL where we see an upregulation of ascorbate, well-established phenotypes in plants. In line with our studies are recent results reported in tomato by Bournonville et al. (2023). Their data corroborated our results in confirming the inhibitory effect of PLP on ascorbate accumulation in tomato. They identified a mutation in the fourth exon of *PLP* in a previously described ascorbate-enriched EMS mutant, P21H6 (Just et al. 2013) as being responsible for the ascorbate buildup in tomato fruits (Bournonville et al. 2023). They further confirmed the interaction between VTC2 and PLP by using BiFC assays in onion cells and heterologous expression in mammalian cells (Bournonville et al. 2023). Similar to our study, they observed a strong interaction between PLP and VTC2 in the dark with this interaction being minimized after blue light exposure. Furthermore, they demonstrated that the blue light-mediated interaction between VTC2 and PLP inhibits GGP enzyme activity (Bournonville et al. 2023). Collectively, our study in Arabidopsis alongside that of Bournonville et al. demonstrates the critical role of PLP in dark-light regulation of ascorbate metabolism. Additionally, our study demonstrates that the cryptochromes CRY1 and CRY2 proteins, other blue light receptors, have putative roles in the light regulation of Arabidopsis.

## Results

### Genome-wide association study identified a novel genetic locus involved in ascorbate accumulation under HL

**Figure 1.**
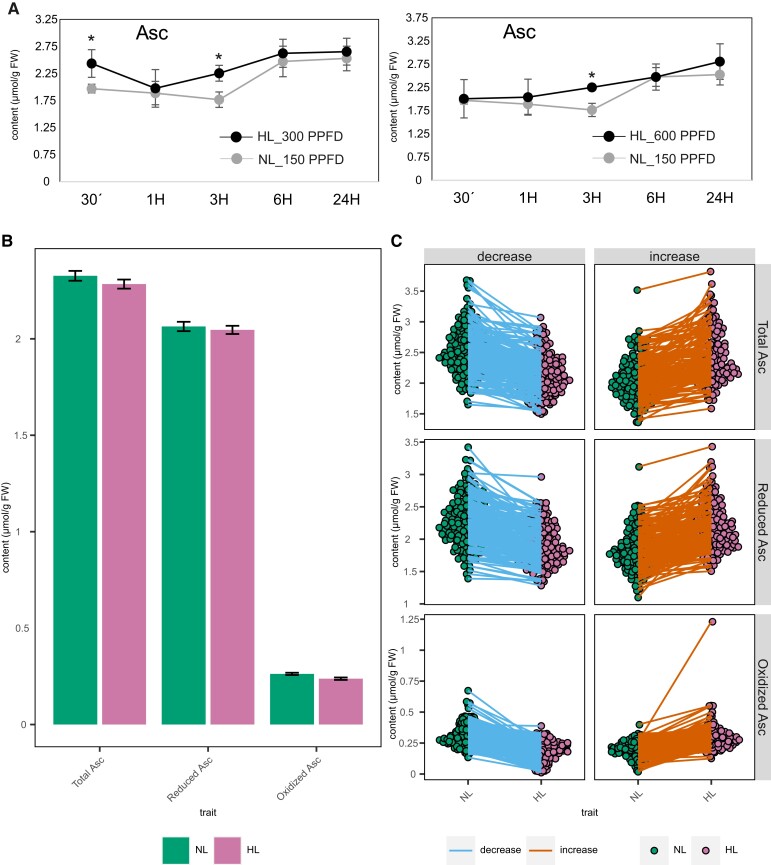
The effect of light intensity on ascorbate of the light acclimation and GWAS experiments. **A)** Total Asc accumulation in *A. thaliana* (Col-0) plants during 24-h light treatments. Plants were grown under short-day conditions at NL (150 *µ*mol m^−2^ s^−1^; PPFD) for 5 wk and transferred to HL conditions (300 and 600 *µ*mol m^−2^ s^−1^; PPFD). Leaves of all the acclimated plants were snap-frozen simultaneously after 30 min, 1, 3, 6, and 24 h in all conditions. Error bars represent Sd (*n* = 4), and asterisks represent significant differences in each time point (*P* < 0.05) calculated using Student's *t*-test. **B)** Arabidopsis accessions were grown as described in **A)** and exposed to HL of 600 *µ*mol m^−2^ s^−1^. After 3 h, leaves of the NL- and HL-acclimated plants were harvested simultaneously. Total and reduced Asc has been measured using the TCEP, HPLC method. DHA levels were calculated by subtracting the reduced from the total Asc. The mean values of all traits are very similar between the NL and HL conditions. Total size for NL = 298; total size for HL = 293. Bars show the mean and Se as error bars. **C)** Different responses of all ecotypes of the population in total, reduced, and oxidized Asc. Each dot represents the Asc content of each ecotype. The total size is described in **B)**. Asc, ascorbate; PPFD, photosynthetic photon flux density; FW, fresh weight.

**Figure 2.**
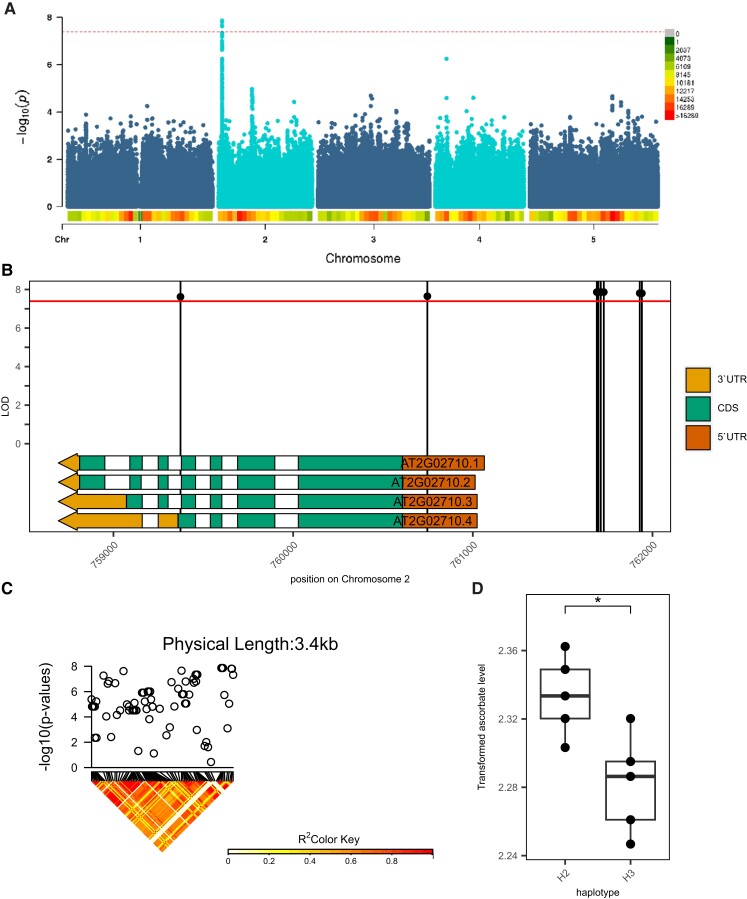
The association found by GWAS between Ascorbate at HL and *PLP*. **A)** Manhattan plot for the total Asc under HL and significant association signals. *P-*values are shown on a log_10_ scale. The *x*-axis shows the physical positions of 5 chromosomes in *A. thaliana*. Significantly associated SNPs pass the Bonferroni threshold, demonstrated by the red line. **B)** The lead SNPs lie upstream of the gene AT2G02710 (*PLP*) and the relative splice variants. In total, 9 significant SNPs above the Bonferroni threshold, of which 7 are located upstream of the gene, 1 in the 5′-UTR region, and 1 in the last exon of the fourth splice variant. **C)** LD of the SNPs located in or close to the *PLP*. The pairwise *R*^2^ values measure LD among all polymorphic sites in *PLP*. The color of each box corresponds to the *R*^2^ value, as shown in the legend. LD was estimated on SNP alleles of 86 SNPs from 285 ecotypes. **D)** Comparative analyses of the content of total ascorbate in the 2 different haplotypes. For both haplotypes, 5 quantiles from 33.3% to 66.6% across the transformed level of ascorbate have been taken, and 5 ecotypes have been selected that matched the quantile levels. The haplotypes show statistically significant different ascorbate levels. Boxplots show the median as the center line and the first and third quartiles as box limits. Whiskers extend as lines outwards from the box until the furthest data point, however with a maximum length of 1.5× in the interquartile range. Statistical significance was estimated with a 2-sided Student's *t*-test and a *P*-value below 0.05 was considered statistically significant and was indicated by an asterisk. *N* = 5 for each haplotype.

To identify novel components of ascorbate signaling or regulation under HL, we employed a GWAS study using the 315-ecotype HapMap population initially assembled by (Li et al. 2010). Several other studies have used these accessions (Wu et al. 2016, 2018; Zhu et al. 2022b). To select the best time-point of light acclimation for the GWAS analysis, preferably aiming at choosing the earliest time point, we performed time-series light acclimation experiments over 24 h (Fig. 1A). We transferred the 5-wk-old Arabidopsis (Col-0) plants grown under 150 *µ*mol m^−2^ s^−1^ light intensity to HL conditions of 300 and 600 *µ*mol m^−2^ s^−1^. We observed that total ascorbate consistently increased after 3 h of HL acclimation in both conditions. Therefore, for our GWAS set-up, we harvested shoots of the normal light (NL, 150 *µ*mol m^−2^ s^−1^) and HL (600 *µ*mol m^−2^ s^−1^) acclimated plants simultaneously after 3 h of light acclimation. Due to the lack of germination of some accessions, we obtained 294 accessions grown in HL and 300 in NL (Supplemental Table S1). We then quantified the total and reduced ascorbate levels by HPLC based on a well-established method (Lykkesfeldt 2000) and subsequently determined dehydroascorbate (DHA) levels by subtracting the reduced ascorbate from that of total ascorbate (Fig. 1B). Ascorbate measurements revealed that the mean values of all traits are not that different, but ecotypes respond differently towards the different light conditions (Fig. 1C). For total ascorbate and reduced ascorbate, we found a highly significant association to a genomic locus on chromosome 2 (Fig. 2A). The lead SNPs lie upstream the gene AT2G02710 which encodes *PLP* (Fig. 2B). In total we found 9 significant SNPs above the Bonferroni threshold of which 7 were found in the region upstream of the gene, one in the 5′-UTR region and one in the last exon of the fourth splice variant. Because many of the SNPs are in high linkage disequilibrium (LD) (Fig. 2C), we limited our further analysis to the 7 highly significant SNPs. Concatenating these 7 SNPs, we found 6 unique haplotypes within the ecotypes under study, which can be grouped into 3 clusters (Supplemental Fig. S1). Most ecotypes reside in haplotypes 2 and 3. For these haplotypes, we took 5 quantiles from 33.3% to 66.6% across the transformed level of ascorbate and selected 5 ecotypes that matched the quantile levels (Fig. 2D). Based on this subsample, the haplotypes show statistically significant different ascorbate levels (Fig. 2D). Since these SNPs were statistically significant only for plants grown in HL conditions, we wondered if haplotypes H2 and H3 (Fig. 2D) are found in geographical areas with distinct levels of solar irradiation. To answer this question, we downloaded data on 6 ecological factors (“UV index spring,” “UV index summer,” “Solar insolation spring,” “Solar insolation summer,” “Net radiation spring,” and “Net radiation summer”) relative to the local environment of 2,999 Arabidopsis accessions were retrieved from AraCLIM database (Ferrero-Serrano and Assmann 2019). The results indicate that accessions with haplotype H2 (that is, having all the alleles associated with higher ascorbic acid levels) are found in environments with considerably lower solar irradiation and UV index than those with haplotype H3 (that is, having all the alleles associated with lower ascorbic acid levels) (Supplemental Fig. S2). These results suggest that Arabidopsis accessions naturally grown under higher solar radiation conditions may have distinct adaptive mechanisms that are not obliged to accumulate higher ascorbate compared to those less exposed to HL. In keeping with this hypothesis is the previous observation made in our laboratory that saiginols, phenylacetylated flavonols, preferentially accumulate in Arabidopsis accessions exposed to HL and high UV irradiance (Tohge et al. 2016). Irrespective of the fact that changes in ascorbate are not paralleled to permanent high-light acclimation, these results indicate that these genome variants are functional for the HL response. It is important to note that the lack of correlation here may be the result of the time-scale of exposure to high-light with our experiments focusing on short-term responses under which ascorbate is known to be highly relevant (Kusano et al. 2011) rather than exposure to high-light permanently during the light-period.

It is worth mentioning that we found 3 other candidate genes in the vicinity of the *PLP* gene with high LOD values, such as AT2G02720 encoding a pectate lyase 6, being involved in the degradation of pectins into D-galacturonate (Uluisik and Seymour 2020) which is a precursor of ascorbic acid in a secondary biosynthetic pathway (Supplemental Fig. S3). AT2G02720 is very close to the leading SNPs (Supplemental Fig. S3). The other 2 genes are AT2G02730 (GRIP/coiled-coil protein, putative (DUF1664), and AT2G02740 (encoding *WHIRLY 3*_*WHY3*; *PLASTID TRANSCRIPTIONALLY ACTIVE11_PTAC11*).

### PLPA and PLPB colocalize with VTC2 and VTC5 in the nucleus and cytoplasm

**Figure 3.**
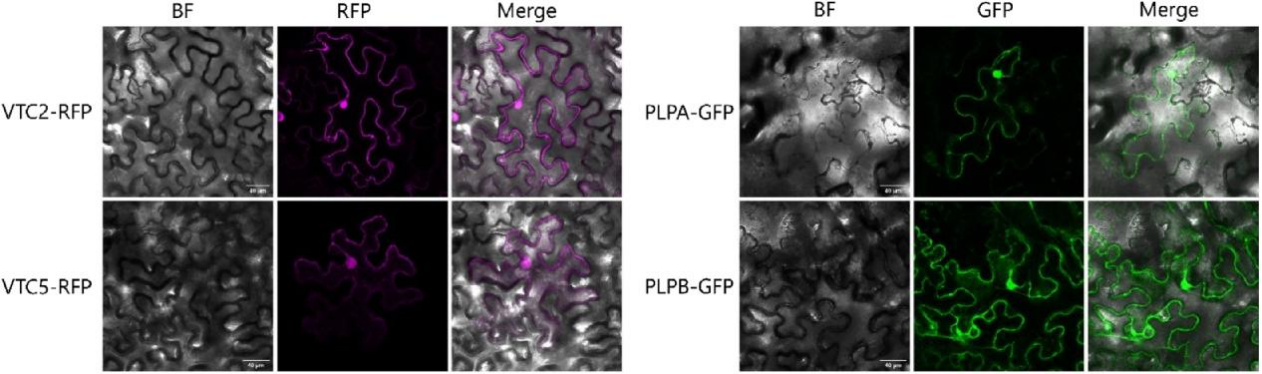
Subcellular localization of PLP and VTC proteins. VTC2 and VTC5 were tagged with RFP at the C-terminal end. PLPA and PLPB were tagged with GFP at the C-terminal end. Eight-week-old *N. benthamiana* plants were transformed withVTC2-RFP, VTC5-RFP (left panel), PLPA-GFP, and PLPB-GFP constructs (right panel). Three days after the agroinfiltration, the infiltrated leaves were subjected to microscopy. Confocal images were taken using a DM6000B/SP5 confocal laser scanning microscope (Leica Microsystems, Wetzlar, Germany). Scale bar = 40 *µ*m. All fusion proteins have a dual localization in the nucleus and cytoplasm. BF, bright field; RFP, red fluorescent protein; GFP, green fluorescent protein; VTC2, VITAMIN C DEFECTIVE 2; VTC5, VITAMIN C DEFECTIVE 5; PLPA, PAS/LOV PROTEIN A; PLPB, PAS/LOV PROTEIN B.

**Figure 4.**
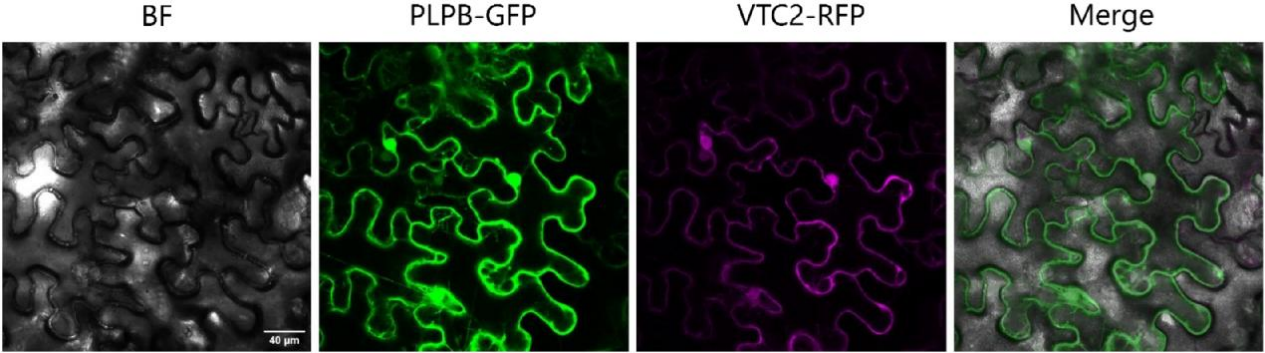
Colocalization of VTC2 and PLPB. Eight-week-old *N. benthamiana* leaves were co-transformed with PLPB-GFP and VTC2-RFP constructs (1:1 ratio). Three days after the agroinfiltration, the infiltrated leaves were subjected to microscopy. Confocal images were taken using a DM6000B/SP5 confocal laser scanning microscope (Leica Microsystems, Wetzlar, Germany). Scale bar = 40 *µ*m. Confocal images show the colocalization of PLPB-GFP and VTC2-RFP in the nucleus and cytoplasm. BF, bright field; RFP, red fluorescent protein; GFP, green fluorescent protein; VTC2, VITAMIN C DEFECTIVE 2; PLPB, PAS/LOV PROTEIN B.

Based on the Araport11 genome release *PLP* has 4 different splice variants, namely *PLPA* (AT2G02710.2), *PLPB* (AT2G02710.1), *PLPC* (AT2G02710.3) and *PLPD* (AT2G02710.4). PLPA (397 amino acids) and PLPB (399 amino acids) isoforms demonstrated 99.5% identity (NCBI, blastP), which were the only isoforms that were shown to interact with VTC2 and VTC5 by Y2H (Ogura et al. 2008b). PLPB has 2 additional amino acids (Ser and Asn) at position 330 (Supplemental Fig. S4), which were proposed to be in the LOV domain (Ogura et al. 2008b). Before analysis of protein–protein interaction between PLP and VTC isoforms, we first investigated the localization of PLPA, PLPB, VTC2, and VTC5 in *Nicotiana benthamiana* leaves. For this purpose, VTC2 and VTC5 fused with RFP at the C-term were expressed in leaves of *N. benthamiana* by agroinfiltration. Consistent with the previous study on the localization of the VTC2 protein (Fenech et al. 2021), we observed a dual localization in the nucleus and cytoplasm for both VTC2 and VTC5 proteins (Fig. 3). Furthermore, the C-terminal GFP fusion of PLPA and PLPB and expression in *N. benthamiana* leaves demonstrated similar localization to VTC2 and VTC5 in the nucleus and cytoplasm (Fig. 3). Moreover, the co-expression of the fusion proteins PLPB-GFP and VTC2-RFP showed that the 2 proteins colocalize (Fig. 4), suggesting they may interact in vivo.

### PLPA and PLPB interact with VTC2, and blue light diminishes their interaction

**Figure 5.**
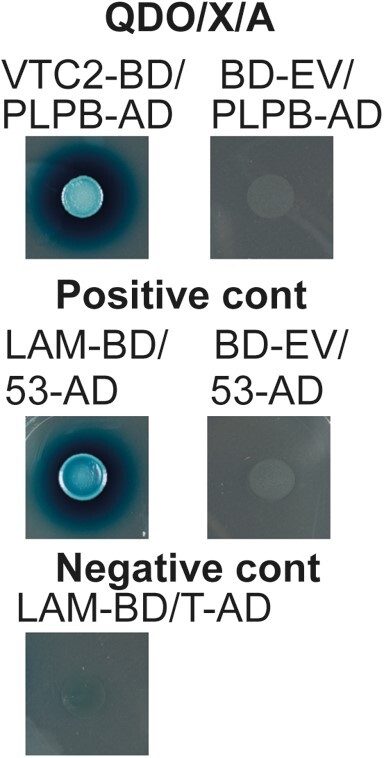
Y2H assay confirming the interaction between VTC2 and PLPB. Y2H was performed using VTC2-BD (as bait) and PLPB-AD (as prey). Yeasts have been grown on -Leu/-Trp/-Ade/-His medium containing X-α-Gal and Aureobasidin (QDO/X/A). Cotransformations of empty bait vector (BD-EV) with PLPB-AD were performed as negative controls (showing no growth). Negative (pGBKT7-Lam and pGADT7-T) and positive (pGADT7-53 and pGBKT7-Lam) controls were used according to the manufacturer's instructions (Clontech).

**Figure 6.**
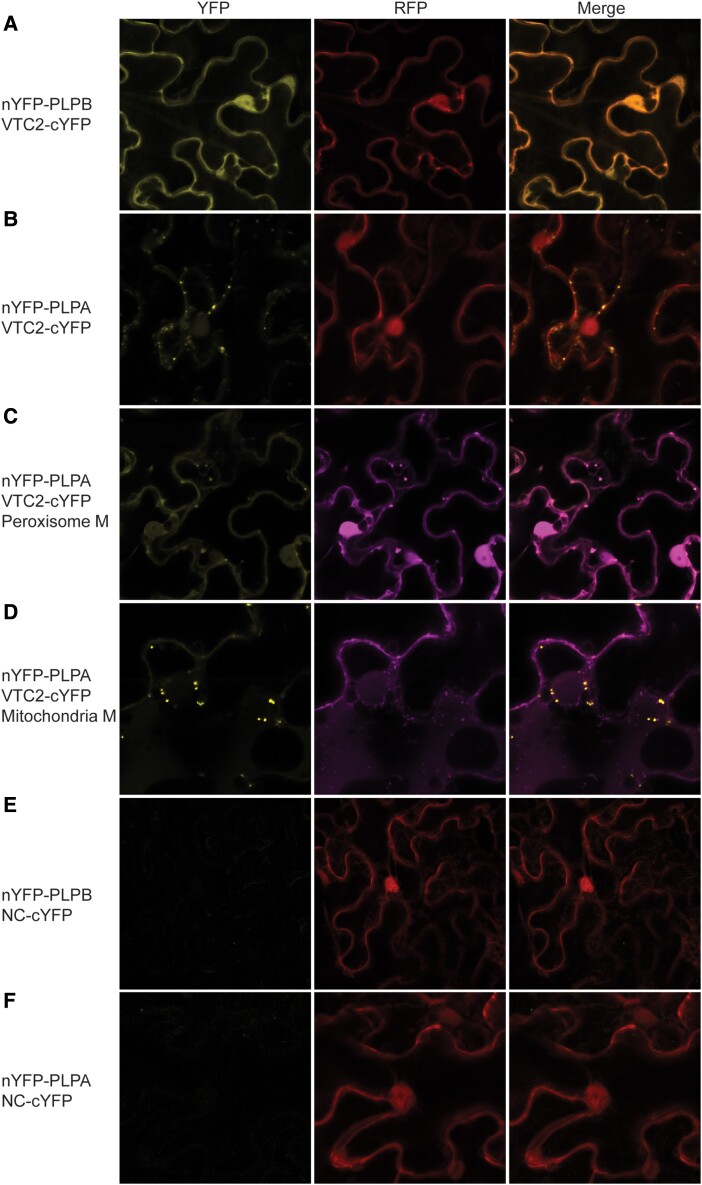
BiFC shows the interaction between VTC2-PLPB and VTC2-PLPA pairs. *N. benthamiana* leaves were transformed with 2in1 BiFC constructs, harboring PLPB-VTC2 **A)**, and PLPA-VTC2 **B)** protein pairs, respectively. A 35S::RFP sequence acts as a positive control for transformation, whereas the YFP signal indicates the physical interaction between the putative interactors. PLPB and VTC2 interact in the nucleus and cytoplasm **A)**. PLPA and VTC2 interact in the nucleus, cytoplasm, and peroxisome **A to D)**. Peroxisome and mitochondria markers (Zhang et al. 2023) fused with RFP were co-transformed with the PLPA-VTC2 pairs into *N. benthamiana* leaves **C** and **D)** respectively. RFP signals of the peroxisome marker overlapped with the YFP signal, confirming the peroxisomal interaction of VTC2 and PLPA **C)**. **E** and **F)** demonstrate negative controls. Scale bar = 11 *µ*m. YFP, yellow fluorescent protein; RFP, red fluorescent protein; VTC2, VITAMIN C DEFECTIVE 2; PLPB, PAS/LOV PROTEIN B; PLPA, PAS/LOV PROTEIN A; M, Marker in Panels **C** and **D**. Mitochondria and peroxisome markers are described in Zhang et al. (2023).

Positive protein–protein interactions between PLPB and VTC2 and PLPB and VTC5 have been reported previously using an untargeted Y2H experiment (Ogura et al. 2008b). To further confirm the interaction between PLPB and VTC2, we first performed a targeted Y2H, using VTC2 as bait (VTC2-BD) and PLPB as prey (PLPB-AD). Y2H demonstrated a strong interaction between VTC2 and PLPB (Fig. 5). To further verify the interactions between PLPB and PLPA with VTC2 in plants that were not studied before, we used the 2 in 1 BiFC vector system (Mehlhorn et al. 2018). BiFC revealed positive interaction between VTC2 and PLPB in the nucleus and cytoplasm (Fig. 6A). Interestingly, PLPA demonstrated positive interaction with VTC2 in the nucleus, cytoplasm, and peroxisome (Fig. 6B). Due to the similarity between mitochondria and peroxisomes, to confirm the localization of the interaction of PLPA and VTC2, we used peroxisome and mitochondria markers fused with RFP and co-transformed with the protein pairs into *N. benthamiana* leaves, respectively (Zhang et al. 2023) (Fig. 6, C and D). RFP signals of the peroxisome marker overlapped with the YFP signal, confirming the peroxisomal interaction of VTC2 and PLPA (Fig. 6C). Furthermore, we did not observe any overlap between the RFP signal of the mitochondrial marker and the YFP signal (Fig. 6D). Therefore, we ruled out the interaction between PLPA and VTC2 to localize in or around mitochondria. In addition, we transformed 2in1 BiFC constructs into *N. benthamiana* leaves, harboring only one protein, PLPB (Fig. 6E), and PLPA (Fig. 6F), respectively serving as controls which we did not observe any YFP signals.

**Figure 7.**
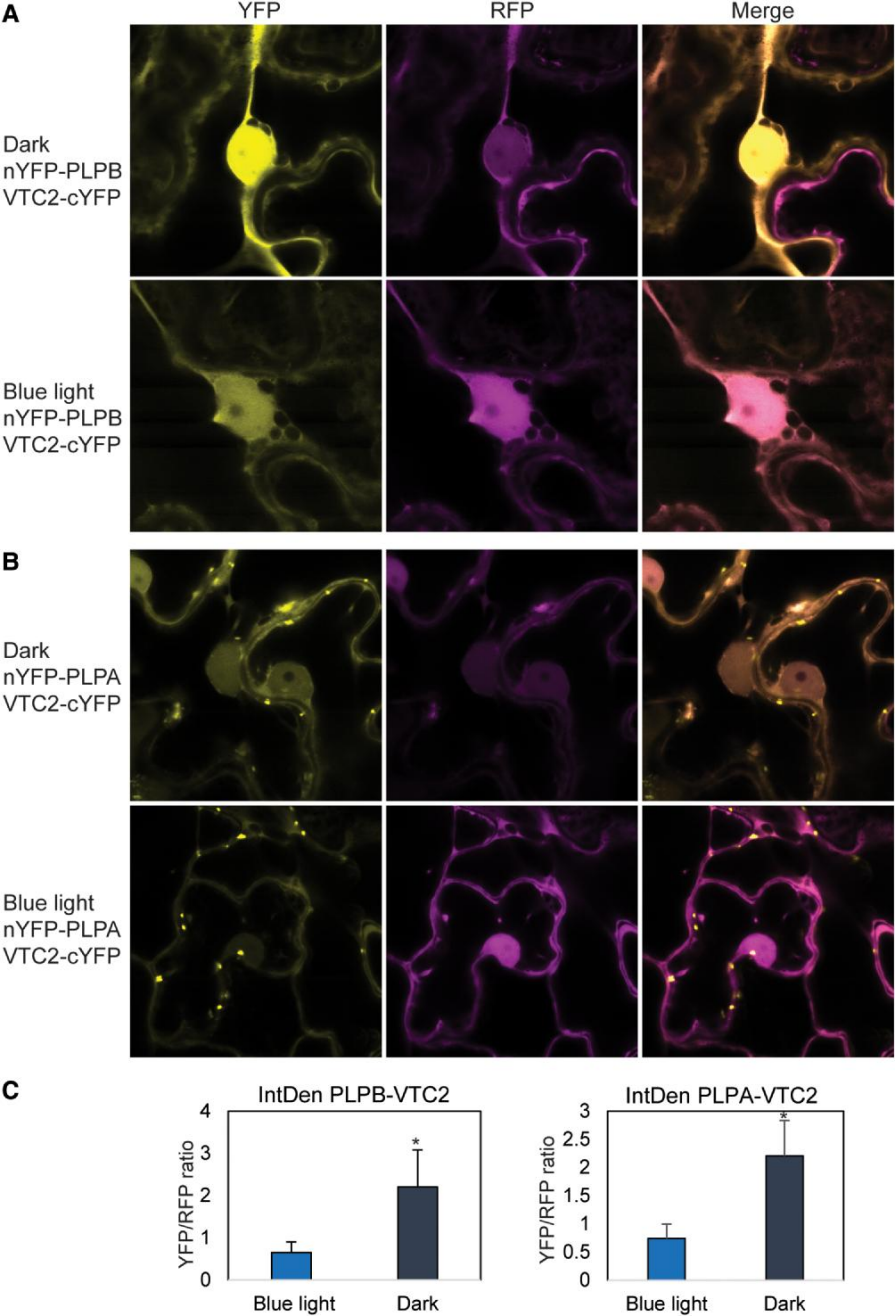
BiFC shows the interaction between VTC2 and PLPA under dark and blue light. *N. benthamiana* leaves transformed with BiFC constructs were exposed to 2 different light conditions: dark and high blue light (300 *µ*mol m^−2^ s^−1^). The YFP signal was stronger under dark conditions than blue light for VTC2-PLPB **A)** and VTC2-PLPA **B)** interactions. Scale bars in A and B = 4.81 *µ*m. **C)** The intensity of YFP and RFP signals in the nuclei of BiFC images was quantified by measuring the integrated density, corresponding to the sum of the values of the pixels in the selected area, using the ImageJ software. Calculating the ratio of YFP/RFP demonstrated that PLPB and PLPA interact more strongly with VTC2 under dark. Error bars represent SD (*n* = 10), and asterisks represent significant differences in each time point (*P* < 0.05) calculated using Student's *t*-test. YFP, yellow fluorescent protein; RFP, red fluorescent protein; VTC2, VITAMIN C DEFECTIVE 2; PLPB, PAS/LOV PROTEIN B; PLPA, PAS/LOV PROTEIN A.

In order to investigate the effect of blue light on VTC2-PLPB and VTC2-PLPA, we performed BiFC in *N. benthamiana* leaves exposed to 2 different light conditions: dark and high blue light (300 *µ*mol m^−2^ s^−1^, Fig. 7). The YFP signal was stronger under dark conditions compared to blue light for both VTC2-PLPB (Fig. 7A) and VTC2-PLPA (Fig. 7B) interactions which is in agreement with previous findings that blue light diminishes the interaction between PLPA and VTC2 in a Y2H experiment (Ogura et al. 2008b). We further quantified the intensity of YFP signals in the nuclei of BiFC images by measuring the integrated density, which corresponds to the sum of the values of the pixels in the selected area using the ImageJ software. Similarly, the integrated density of the RFP signals of the same chosen areas of the YFP signals, which lay in the backbone of the 2 in 1 BiFC vector, were quantified. Calculating the ratio of YFP/RFP demonstrated that PLPB and PLPA interact more strongly with VTC2 under dark (Fig. 7C), and blue light significantly diminished the interaction of the 2 protein pairs (Fig. 7C).

### *plp* Mutants demonstrated higher ascorbate levels at different light qualities

To gain further insight into the function of PLPB in ascorbate biosynthesis, we isolated 2 homozygous T-DNA insertion lines (*plp1*, *plp2*) and selected one line of *plp1* and 2 lines of *plp2* (*plp2-1*, *plp2-2*) for further analyses. Mutants and WTs (Col-0) were grown under constant light conditions on agar plates for 3 wk. The mutants did not have any phenotypic changes compared to the WT. However, ascorbate measurement revealed a significant increase of 1.5- to 1.8-fold higher ascorbate levels in the *plp* mutants relative to WTs, suggesting an inhibitory role for PLP in the regulation of ascorbate (Supplemental Fig. S5A). The *plp* mutants contained 7% to 10% of WT expression levels and demonstrated a significant increase in *VTC2* expression levels (Supplemental Fig. S5B), which agrees with our observation regarding the higher ascorbate levels of the *plp* mutants.

**Figure 8.**
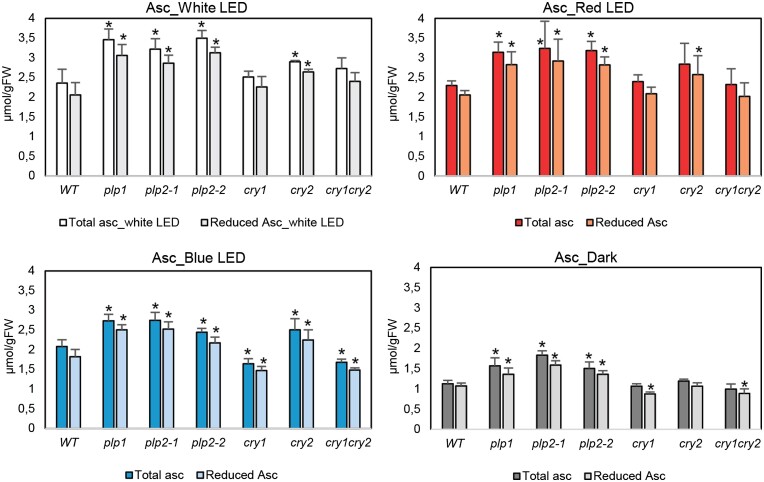
Ascorbate levels of WT Arabidopsis and *plpb* and cry mutants exposed to different light qualities. The mutants and (WTs; Col-0) were grown under long-day growth chambers equipped with white LED lights (126 *µ*mol m^−2^ s^−1^) for 5 wk. Then plants were transferred simultaneously into red (LED lights, 126 *µ*mol m^−2^ s^−1^), blue (LED lights, 126 *µ*mol m^−2^ s^−1^), and dark conditions for 3 d. Rosettes were harvested and used for ascorbate measurements. Error bars represent Sd (*n* = 5), and asterisks represent significant differences between the mutants and the corresponding WT at each light condition, calculated using Student's *t*-test (*P* < 0.05). Asc, Ascorbate; FW, fresh weight; LED, light emitting diode.

To further investigate the regulatory role of PLPB on ascorbate in response to light spectra, we grew the mutants and WTs under long-day conditions in soil equipped with white LED lights (126 *µ*mol m^−2^ s^−1^). We also included the blue receptor cryptochromes, *cry1*, *cry2*, and *cry1cry2*, in this experiment to assess the specificity of PLPB role in ascorbate regulation in response to the blue light. After 5 wk under white light, we transferred them into chambers with exclusively red and blue LED lights with an equal light intensity of 126 *µ*mol m^−2^ s^−1^, respectively. We also transferred one-quarter of the white light-grown plants into a dark chamber. Finally, after 3 d of being under different light conditions, we harvested the whole rosettes and measured the total and reduced ascorbate pools. As shown in Fig. 8, all *plp* mutants demonstrated a significant increase in ascorbate levels relative to WT under all light qualities. Furthermore, the fold changes of ascorbate compared to WT were comparative in all light conditions, regardless of light quality (Supplemental Fig. S6). These results demonstrate that PLPB does not negatively impact ascorbate levels exclusively under blue light. Furthermore, we could observe that similar to *plp*, the *cry2* mutant contained slightly but significantly higher ascorbate levels than WT under white, red, and blue light conditions (Fig. 8). Moreover, the *cry1* mutant significantly reduced ascorbate levels under blue light and dark (Fig. 8). These results suggest CRY1 and CRY2 as other regulators of ascorbate which act antagonistically in regulating ascorbate at different lights, especially under blue and dark (Fig. 8). Furthermore, we observed that *plp* and *cry1* mutants had significantly higher DHA levels under the dark than WT (Fig. 8B), suggesting a putative role for these proteins in the regeneration of ascorbate in the dark.

## Discussion

### Role of ascorbate in light acclimation and the involvement of PLP proteins

Plants have developed rapid and late acclimation responses to environmental fluctuations. Light acclimation entails metabolic adjustments to maintain overall plant fitness and avoid photodamage (Dietz 2015). Metabolic responses per se occur in response to substantial transcriptome reprograming (Huang et al. 2019; Garcia-Molina et al. 2020; Zirngibl et al. 2023). Ascorbate with vital antioxidant properties for ROS detoxification under HL stress has profound bidirectional relationships with the rates of photosynthesis and mitochondrial electron transport chain (Mano et al. 2004; Nunes-Nesi et al. 2005; Tóth et al. 2009, 2011); is, therefore, considered as an essential metabolic modulator under HL acclimation. However, ascorbate has been shown to increase on longer time scales of light acclimation ranging from 3 h to days (Page et al. 2012; Alsharafa et al. 2014; Rosado-Souza et al. 2020). In accordance with these observations, our study demonstrates that ascorbate consistently increases after 3 h of HL acclimation by exposing the plants to 300 and 600 HL intensities. Despite the importance of ascorbate under HL, and considering the fact that VTC2, the most controlling enzyme of the ascorbate biosynthesis pathway, demonstrated a translational regulatory mechanism in balancing the ascorbate levels (Laing et al. 2015) and, most importantly, it controls the ascorbate pools transcriptionally and enzymatically in response to light, there is a knowledge gap on how this enzyme is regulated by light.

Metabolic GWAS has been used as a powerful approach in mining genes or genetic markers associated with relevant agronomic traits and metabolites for multiple plants such as Arabidopsis, rice, maize, soybean, and tomato (Wu et al. 2016; Fang et al. 2017; Fang and Luo 2019; Zhu et al. 2022b). Moreover, this approach has been implemented in biotic and abiotic stress studies to estimate genetic associations of the stress response (Zhu et al. 2022a). Given the power of this approach in our study, we identified highly significant SNPs in chromosome 2, demonstrating strong associations with total and reduced ascorbate pools under HL. In addition, leading SNPs above the Bonferroni threshold revealed *PLP* as the determinant of ascorbate levels under HL. Moreover, we could not obtain any significant SNPs under NL, demonstrating the importance of this gene in high-light acclimation. Furthermore, most of the significant SNPs were located upstream of *PLP*, suggesting that *PLP* expression levels are critical in controlling the ascorbate levels, similar to *VTC2*, which is controlled at the expression levels under HL (Dowdle et al. 2007; Yabuta et al. 2007; Müller-Moulé 2008; Page et al. 2012; Schmitz et al. 2014; Laing et al. 2017). In agreement with this, Li et al. (2018b) demonstrated that *Glycine max PAS/LOV PROTEIN1* (*GmPLP1*) expression levels were significantly affected by light, especially blue light. Therefore, it was proposed as a putative blue light receptor (Li et al. 2018b).

Analyzing the 5 statistically significant SNPs for ascorbate levels in HL, we discovered that they are in high LD and that the accessions having the haplotype associated with higher ascorbic acid levels are found in local environments with lower UV index and solar insolation, supporting the hypothesis that these genome variants are indeed functional for the response to HL. Furthermore, the fact that alleles associated with higher ascorbic acid levels are more frequent in ecosystems with lower solar radiation suggests an adaptive/evolutionary mechanism for the light-regulation of ascorbate in plants. As previous work in our lab has shown accessions that are naturally more exposed to HL possess other protective mechanisms against HL (Tohge et al. 2016), therefore they do not need to upregulate ascorbate levels for HL acclimation as much as accessions that are less exposed to the sun in their native habitat.

### Dark-light regulation of ascorbate

Besides the highly significant SNPs demonstrated the relevance of PLP protein to ascorbate levels explained above, 2 previous studies prompted us to further explore the molecular mechanism of PLP proteins in more detail. First, Ogura et al. (2008b) demonstrated that PLPA interacts with VTC2 and VTC5 in yeast. Second, Li et al. (2018b) proposed PLP as a putative blue light receptor after detecting higher Gm*PLP* expression under blue light. In this study, we first identified that all PLPA, PLPB, VTC2, and VTC5 proteins are localized to the nucleus and cytoplasm, suggesting the possibility of their interaction. Then, using BiFC, we confirmed the interaction between PLPA and VTC2 and also between PLPB and VTC2.

Furthermore, in agreement with the previous study on the diminishing effect of blue light on VTC2-PLPA (Ogura et al. 2008b), our BifC studies demonstrated much stronger interactions between VTC2-PLPA and VTC2-PLPB protein pairs under the dark compared to blue light. Moreover, ascorbate measurements of the *plp* knock-down mutants demonstrated that PLPB is an inhibitor of ascorbate metabolism, as the *plp* mutants showed higher ascorbate levels than WT. We did not observe any specific response to blue light in ascorbate accumulation as *plp* mutants had similar higher ascorbate levels at all single light spectra tested. This observation suggests the involvement of other putative regulators in the blue light regulation of ascorbate. PLPB might also be vital in red light perception as the mutants had higher ascorbate levels than WT. However, *plp* mutants demonstrated an almost 3-fold increase in the levels of DHA under the dark, demonstrating that the regeneration of ascorbate may have perturbed under the dark, either dependent or independent of *plp*. It has also been observed that *PLPB* expression increases strongly under dark conditions (Li et al. 2018b), pointing to its vital regulatory role in the dark.

Generally, these results delineate the underlying mechanism controlling the dark-light regulation of ascorbate. Our data suggest that PLP inhibits VTC2 in the dark by direct protein binding. Therefore, ascorbate is strongly reduced, whereas this interaction is released under the light, presumably by a conformational change of the PLP protein after sensing the blue light spectrum. Therefore, ascorbate levels are increased under light compared to dark conditions. Bournonville et al. (2023) used in vitro activity assays as a means to investigate the inhibitory effect of PLP on the GGP enzyme. They demonstrated that the blue light-mediated interaction between PLP and VTC2 inhibits GGP enzyme activity. Kinetic experiments revealed that PLP returns to its active “dark” form very slowly following exposure to blue light. Further studies are, however, needed to investigate whether this negative effect on enzyme activity is promoted through protein degradation.

Furthermore, PLP has a distinct PAS/LOV domain compared to the phototropins, PHOT1 and PHOT2, which have 2 tandem LOV domains (Kasahara et al. 2010). The LOV2 domain of phototropins contains a conserved cysteine residue that, upon blue light illumination, binds to 2 FMN chromophores and reverses under dark conditions (Paik and Huq 2019). Similar to the phototropins, the PLP protein has such a conserved domain (UniProt; Ogura et al. 2008a, Kasahara et al. 2010), therefore, we assume similar conformational switches under dark-light conditions for PLP proteins.

It is worth mentioning, in our study, expression analysis of the *plp* mutants demonstrated a remarkable up-regulation in *VTC2* expression. Given the post-translational effect of PLP on VTC2, the overexpression of *VTC2* in *plp* mutants is somewhat puzzling. This observation may indicate that either VTC2 controls its own expression in a positive feedback loop or that PLP inhibits the expression of *VTC2* by direct binding to the *VTC2* promoter. The latter hypothesis is, however, unlikely given the lack of DNA-binding domains in VTC2 and PLPB. That said, the nuclear localization of both proteins renders it difficult to formally exclude this possibility and the possibility that PLPB inhibits other activators of *VTC2* suggests that further research is required to clarify this issue. Our study also demonstrates that PLPA and VTC2 interact in the peroxisome, suggesting a new site for the metabolism or regulation of ascorbate in plant cells requiring further investigations. Furthermore, it has been demonstrated that among all cellular compartments, the peroxisomes contain the highest levels of ascorbate, 23 mM, which is almost twice as high as that found in chloroplasts (Zechmann 2018). Peroxisomes have fundamental roles in plant metabolism as they are the site of multiple processes, including photorespiration in leaves, and biosynthesis of some hormones such as indole-3-acetic acid, jasmonic acid, and salicylic acid (Su et al. 2019). Furthermore, these organelles are not isolated and are in direct communication with other organelles, such as mitochondria and chloroplasts to execute their signaling roles such as photorespiration, and redox metabolism (Shai et al. 2016; Su et al. 2019). Moreover, peroxisomes can sense ROS/redox changes in response to environmental stresses and trigger rapid responses such as ROS-dependent signaling networks (Sandalio and Romero-Puertas 2015; Pan et al. 2020). Additionally, ascorbate displays a specific subcellular distribution pattern in response to HL (Heyneke et al. 2013). When Arabidopsis plants were exposed to 700 *µ*mol m^−2^ s^−1^ light intensity, the ascorbate content of most cellular compartments was increased, however, the ascorbate content of the peroxisome was remarkably reduced (Heyneke et al. 2013). Furthermore, *vtc2* mutants were shown to display 166% higher ascorbate levels in their peroxisomes compared to WT after exposure to HL for 14 d (Heyneke et al. 2013). Therefore, ascorbate in peroxisomes appears to be essential for plant protection against HL stress (Zechmann 2018).

### Other putative regulators of ascorbate biosynthesis

In this study, we identified CRY1 and CRY2 as other regulators of ascorbate which act antagonistically in regulating ascorbate at different lights, especially under blue and dark. Like *plpb*, the *cry2* mutant contained higher ascorbate levels than WT under white, red, and blue light conditions. Whereas the *cry1* mutant significantly reduced ascorbate levels under blue light and dark. Therefore, it appears that CRY1 is a positive regulator of ascorbate under dark and blue lights, and CRY2 is a negative regulator of ascorbate. Cryptochromes are other blue light receptors with multiple regulatory functions in the plant life cycle (Wang and Lin 2020). Alternate signaling for cryptochromes has been proposed because it has been shown that cryptochromes also synthesize ROS in response to light (El-Esawi et al. 2017) which might be linked to ascorbate signaling. Furthermore, it has been revealed that CRY1 is essential for the induction of photoprotective mechanisms in response to HL (Kleine et al. 2007). Further studies are required to clarify the molecular link between the CRY proteins and VTC2. Moreover, the magnitude of ascorbate changes in *plpb* and *cry2* knockouts is not massive, demonstrating that other negative regulators have roles in the dark-light regulation of ascorbate. As described in the introduction, CSN5B and AMR1 are other negative regulators of ascorbate in the dark (Zhang et al. 2009; Wang et al. 2013).

Our GWAS also suggests other putative genes in ascorbate signaling under HL conditions, such as a pectate lyase 6, which is involved in the degradation of pectins into D-galacturonate, is a precursor of ascorbic acid in a secondary biosynthetic pathway (Uluisik and Seymour 2020). Another putative candidate is *WHIRLY 3*_*WHY3*; *PLASTID TRANSCRIPTIONALLY ACTIVE11_PTAC11*, a close homolog of *WHIRLY 1* (78% identity). WHYRLY1 forms chloroplast oligomers responsible for plastome organization and stabilization (Cappadocia et al. 2012). Foyer et al. proposed that, upon stresses like HL and pathogen infection, the oligomers disassemble to form monomers, probably through a mechanism that involves redox-sensitive cysteines. In this way, monomers are able to migrate to the nucleus to change gene expression (Ströher and Dietz 2008; Foyer et al. 2014). These findings may pave the way for future research.

## Conclusions

In this study, we have used GWAS to delineate the genetic factors that underlie increased ascorbate accumulation in Arabidopsis during acclimation to high-light stress. Most prominent amongst these was the strong association between ascorbate levels and HL and the region upstream and within the PAS/LOV protein (*PLP*) gene. Intriguingly, the PLPA and PLPB splice variants co-localized with VTC2 and VTC5 in both the cytosol and nucleus. Furthermore, yeast 2-hybrid and BiFC analyses revealed that PLPA and PLPB interact with VTC2, and that blue light inhibits this interaction. Furthermore, PLPB knockout mutants were characterized by 1.5- to 1.7-fold elevations in their ascorbate levels. Our results thus collectively indicate the PLP plays a critical role in the elevation of ascorbate levels which are a signature feature of the short-term HL acclimation response. Future studies should focus on dissecting the molecular hierarchy of the control of ascorbate content under a range of other environmental conditions.

## Materials and methods

### Plant material and growth conditions

All *A. thaliana* WT and mutant lines were in the Columbia-0 (Col-0) background, except for the GWAS experiment, where around 300 accessions were grown (Supplemental Table S1). Seeds were sown directly on the soil, and plants were raised in a growth chamber in the following conditions unless otherwise specified: 150 *µ*mol m^−2^ s^−1^ light intensity, 40% relative humidity, 22°C temperature, 8/16-h light/dark cycles (short-day). HL (300/600 *µ*mol m^−2^ s^−1^) treatments were applied with the same growth conditions after being 5 wk old for the duration of 3 d. For the light spectra plate and soil experiments, homozygous *plpb* T-DNA insertion lines (*plp1*: SALK-031071_N531071 and *plp2*: SALK_066978C_ N675614) were screened by primers as in Supplemental Table S2. Cryptochrome mutants were kindly provided by Prof. Alfred Batschauer: *cry1*, *cry2*, *cry1cry2 (*Mockler et al. 1999*)*. For the plate experiment, seeds were sterilized and sown on ½ MS media, supplemented with 1% (w/v) sucrose, and grown for 3 wk under a chamber equipped with white light LEDs (constant light 125 *µ*mol m^−2^ s^−1^; 20°C temperature). For the light spectra soil experiment, plants were grown under white LED, 126 *µ*mol m^−2^ s^−1^ light intensity, 40% relative humidity, 22°C temperature, and 16/8-h light/dark cycles (long-day). Five weeks-old plants were transferred to chambers with similar growth conditions except for the light conditions, blue (440 nm), red (640 nm), and dark. *N. benthamiana*, WT plants, were grown under short-day conditions (8 h light/16 h dark), 100 *µ*mol m^−2^ s^−1^ light intensity, 21°C/16°C day/night temperature, and 60% humidity.

### HPLC measurements of ascorbic acid

Total ascorbate pool (Asc) and DHA were determined by HPLC following a modified protocol from (Lykkesfeldt 2000). Firstly, the entire rosettes were excised and snap-frozen in liquid nitrogen. For each sample, 50 mg of ground frozen tissue was aliquoted. After adding 500 *µ*L of extraction buffer (EDTA 2 mM, orthophosphoric acid 5% v/v), each sample was immediately vortexed for 30 s and kept on ice. Subsequently, samples were centrifuged at 4°C for 30 min at 16,000 × *g*. Then, 200 *µ*L of the supernatants were aliquoted in 2 different sets. 200 *µ*L extraction buffer was added to the first set to measure the reduced Asc. 200 *µ*L of freshly prepared extraction buffer with TCEP (EDTA 2 mM, orthophosphoric acid 5% v/v, TCEP 10 mM) were added to the second set to reduce DHA to Asc. Samples were left for 15 min at room temperature to allow the TCEP-mediated reduction of DHA to Asc reduction and were centrifuged immediately after at 4°C for 5 min at 16,000 × *g* to pellet potential impurities. 100 *µ*L of assay mix were then aliquoted in vials and kept at 4°C before HPLC measurements. Two mobile phases were used: Buffer A (KH_2_PO_4_ 50 mM, pH 2.5) and Buffer B (100% (v/v) acetonitrile). Twenty microliters of assay mix were injected onto a Eurospher column (KNAUER, 100-5 C18, 5 *µ*m, 250 mm × 4 mm) and subjected to the following gradient using an UltiMate 3000 HPLC system (Dionex, Sunnyvale, CA, USA): 100% (v/v) Buffer A for 3.5 min, 70% (v/v) Buffer A and 30% (v/v) Buffer B for 5 min, and additional 5 min using 100% Buffer A. Flow rate was maintained at 1 mL min^−1^ and the assay was carried out at 4°C. Ascorbic acid was detected using an UltiMate 3000 Diode Array detector (Dionex, Sunnyvale, CA, USA) at 244 nm. Data were analyzed with Chromeleon software (Dionex, Sunnyvale, CA, USA). Ascorbic acid was quantified by comparison with standards using the peak areas at 244 nm.

### Genome-wide association study

A genome-wide association study (GWAS) was performed using R software (R Core Team 2020) for a total of 12 traits: total ascorbate pool, reduced ascorbate, DHA, and DHA/Asc in NL, HL and HL/NL. All data were BoxCox-transformed using the function powerTransform within the package car (Fox 2019). Genotypic data was downloaded from Arouisse et al. (2020), Variant Call Format (vcf), and converted to HapMap (hmp) format using TASSEL software. Genome-wide association analysis was performed using 1.242.079 SNPs for NL conditions and 1.233.912 for HL conditions, with minor allele frequency > 5%. The Efficient Mixed Model Association algorithm was used to estimate variance components (VanRaden 2008), and the model was fitted using the function MVP of package rMVP (Yin et al. 2021). LD heatmap was created with the LDheatmap package in R (Shin et al. 2006). Haplotype distance was calculated with the DistanceMatrix function of the DECIPHER package using Hamming's distance (Wright 2016). Haplotypes were clustered by the ward's minimum variance method. Other analysis and visualization were done with base R and the help of the following packages: tidyverse, gggenes, ggpubr, ggplotify, cowplot, and tidyverse (Wickham et al. 2019). All the genotype and geoclimatic data were downloaded from open-access databases and analyzed using a combination of statistical packages provided by the RStudio software. Six ecological factors (“UV index spring,” “UV index summer,” “Solar insolation spring,” “Solar insolation summer,” “Net radiation spring,” and “Net radiation summer”), relative to the local environment of 2,999 Arabidopsis accessions were retrieved from AraCLIM database (Ferrero-Serrano and Assmann 2019). The SNP matrix relative to 1,135 Arabidopsis accessions was downloaded directly from the website of the 1001 Genome Project (Weigel and Mott 2009).

### Cloning

For the subcellular localization of PLPA, PLPB, VTC2, and VTC5, the coding sequences lacking the stop codon were cloned using the Gateway system (Invitrogen). The BP recombination was performed to clone each coding sequence into the pDONR207 entry vector (Invitrogen). Subsequently, LR recombination was performed to clone the coding sequences of PLPA and PLPB into the pK7FWG2 (Karimi et al. 2002) destination vector and the coding sequences of *VTC2* and *VTC5* into the pUBC-RFP-DEST (Blatt and Grefen 2014) destination vector.

For BiFC, the coding sequences containing the stop codon of *PLPA*, *PLPB* were cloned into the pDONR221 P2-P3 entry vector (Invitrogen), whereas the coding sequences lacking the stop codon of *VTC2* were cloned into the pDONR221 P1-P4 entry vector (Invitrogen). LR recombination was always performed using the pBiFCt-2in1-NC as the destination vector (Mehlhorn et al. 2018).

For the Y2H assays, the coding sequences containing the stop codon of *PLPB* were cloned using the In-Fusion HD Cloning Kit (Takara Bio) following the instructions of the kit user manual. Briefly, the pGADT7 vector (for the prey; Clontech) was cut using the restriction enzymes EcoRI and SmaI and the pGBKT7 vector (for the bait; Clontech) was cut using EcoRI and SalI. All restriction enzymes employed here were FastDigest Restriction Enzymes (ThermoFisher Scientific) and the restriction digestion was performed following manufacturer's instructions. A PCR was performed with gene-specific primers containing the same restriction sites which had been digested in the vectors to be cloned. Subsequently, the recombination between the linearized vector and the purified PCR product was performed using the In-Fusion HD Enzyme (Takara Bio). The pGADT7 AD and pGBKT7 vectors containing the coding sequence of *VTC2* were kindly provided by Prof. Miguel A. Botella (Fenech et al. 2021). Primers for all the clonings described above are listed in Supplemental Table S2.

### Agrobacterium transformation and agroinfiltration

Agrobacterium transformation was performed as described by Zhang et al. (2020). Briefly, *Agrobacterium tumefaciens* strain AGL1^20^ was cultured overnight in YEB medium with carbenicillin 25 mg L^−1^ and rifampicin 20 mg L^−1^ at 28°C. The agrobacteria were subjected to 3 sequential steps of centrifugation (30 s, 20,000 × *g*, 4°C) and washing with 1 mL, 500 *µ*L, and finally 200 *µ*L of ice-cold water. Around 1 *µ*g of vector DNA was added into a 2-mL tube with 45 *µ*L of competent cells and incubated on ice for 5 min. After electroporation at 1.8 kV, transformed agrobacteria were shaken at 250 rpm in 1 mL YEB medium for 1 h at 28°C. Afterward, cells were plated on a YEB plate (Carb+, Rif+, and appropriate antibiotics) and incubated at 28°C for 2 to 3 d. Then, agrobacteria were transferred to a plate containing a modified YEB medium (yeast extract 1.0 g L^−1^, Beef extract 5.0 g L^−1^, peptone 5.0 g L^−1^, sucrose 5.0 g L^−1^, MgSO_4_·7H_2_O 0.5 g L^−1^, agarose 1%, NH_4_Cl 1 g L^−1^, KCl 0.15 g L^−1^, CaCl_2_ 0.01 g L^−1^, FeSO_4_·7H_2_O 0.0025 g L^−1^, 50 mM phosphate buffer 40% v/v, 20% glucose 50% v/v, 1 M MES 20% v/v, 200 *µ* M acetosyringone 1% v/v, pH 5.5) and incubated at 28°C for 2 to 3 d.

Cells were scraped and resuspended in 500 *µ*L of washing solution (10 mM MgCl_2_, 100 *µ* M acetosyringone). After a quick vortex, the OD600 was measured and agrobacteria were diluted to a final OD600 of 0.5 in infiltration solution (¼MS at pH 6.0, 1% (w/v) sucrose, 100 *µ* M acetosyringone, 0.005% v/v Silwet L-77). The obtained solution was infiltrated in leaves of 8-wk old *N. benthamiana* using a 1-mL plastic syringe and they were kept in the dark overnight at room temperature. The transformed plants were then transferred back to normal growth conditions (unless otherwise specified) for another 2 to 3 d before microscopy.

### Microscopy

All confocal images were taken using a DM6000B/SP5 confocal laser-scanning microscope, and 63× water objective was used (Leica Microsystems, Wetzlar, Germany). For images depicted in Fig. 6, 514 nm laser at 10% and 569 nm laser at 10% were used for YFP, and RFP signals, respectively. Emission ranges were collected between 519 and 558 nm with a gain of 697 V for YFP signals and between 568 and 625 nm with a gain of 637 V for RFP signals. For images depicted in Fig. 7, 514 nm laser at 40% and 561 nm laser at 20% were used for YFP, and RFP signals, respectively. Emission ranges were collected between 520 and 565 nm with a gain of 643 V for YFP signals and between 575 and 640 nm with a gain of 755 V for RFP signals.

### Y2H assay

*Saccharomyces cerevisiae* strain Y2HGold (Takara Bio) was transformed with the constructs described in the “Cloning” section using the lithium-acetate transformation method (Gietz and Woods 2006). Transformants were grown on a plasmid-selective medium (synthetic defined (SD)/-Trp-Leu) and grown at 30°C for 3 to 5 d. Two independent colonies were taken per transformation event and resuspended in 120 *µ*L of sterile water. Yeast growth was spotted on both a plasmid-selective medium (SD-Trp-Leu) and an interaction-selective medium consisting of an SD-Trp-Leu-His-Ade medium with the addition of Aureobasidin A (AbA, 0.2 *µ*g mL^−1^) and X-α-Gal (20 mg mL^−1^). Plates were left at 30°C for 5 d before pictures were taken.

### Accession numbers

Sequence data from this article can be found in the GenBank/EMBL data libraries under accession numbers AY058232, AF508793, and AY091285 for *PLP*, *VTC2*, and *VTC5*, respectively.

## Supplementary Material

kiad323_Supplementary_Data

## Data Availability

Data supporting this study are included within the article and/or supporting materials. The authors responsible for providing more information upon request are Fayezeh Aarabi and Alisdair Fernie.

## Supplemental data

The following materials are available in the online version of this article.

**Supplemental Figure S1.** GWAS haplotype groups based on significant SNP markers.

**Supplemental Figure S2.** Association of SNP haplotypes with the local environment of Arabidopsis accessions.

**Supplemental Figure S3.** Position of the SNPs identified in GWAS for total Asc under HL.

**Supplemental Figure S4.** Protein alignment of PLPB and PLPA.

**Supplemental Figure S5.** Ascorbate and gene expression levels of the *plpb* knockouts grown on agar plates.

**Supplemental Figure S6.** Heatmap demonstrating the ascorbate levels of WT and the mutants exposed to different light qualities.

**Supplemental Table S1.** GWAS ecotypes used in this study and the corresponding ascorbate levels under normal and HL conditions.

**Supplemental Table S2.** List of primers used in this study.
