# Supplementary material for: Genome-wide association study unveils ascorbate regulation by PAS/LOV PROTEIN during high light acclimation
Source: Plant Physiol. 2023 Jun 2;193(3):2037–54. doi: 10.1093/plphys/kiad323 (PMC10602610; doi:10.1093/plphys/kiad323)
Supplement: kiad323_Supplementary_Data [file kiad323_supplementary_data.zip › Supplemental data.pdf]

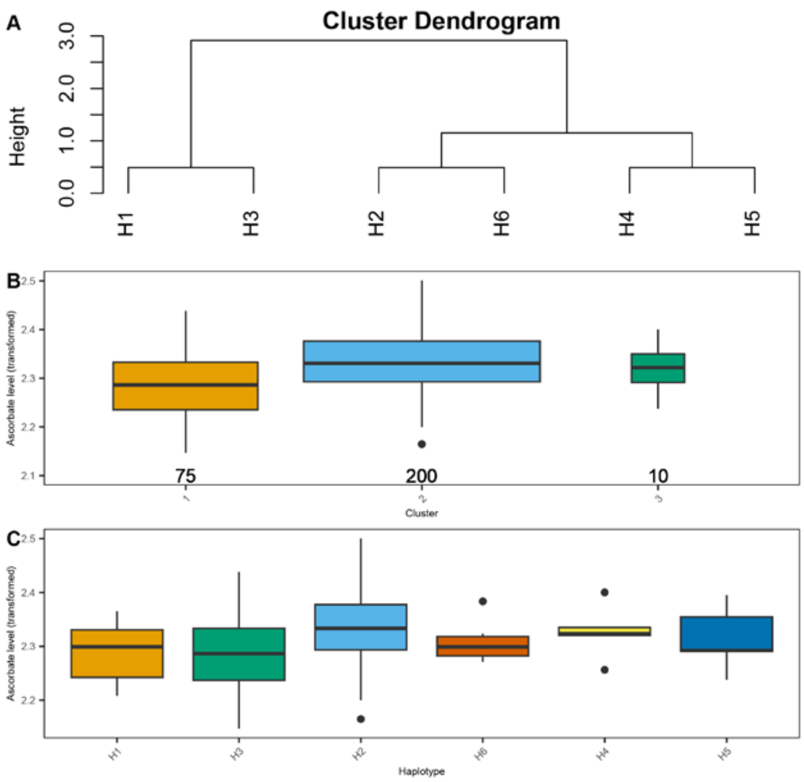

Supplemental Figure S1. GWAS haplotype groups based on significant SNP markers. (A) Sequence distance between haplotype groups as estimated by Hamming's distance and clustered by ward's minimum variance method. (B) Level of transformed ascorbate values of haplotype clusters, grouped based on their distance in A. Numbers below boxplots indicate the number of ecotypes included in the respective clusters. (C) Level of transformed ascorbate values in individual haplotypes. Boxplots show the median as the center line and the 1st and 3rd quartile as box limits. Whiskers extend as lines outwards from the box until the furthest data point, however with a maximum length of 1.5x the interquartile range (IQR). Points beyond 1.5x the IQR are displayed as individual outlier dots.

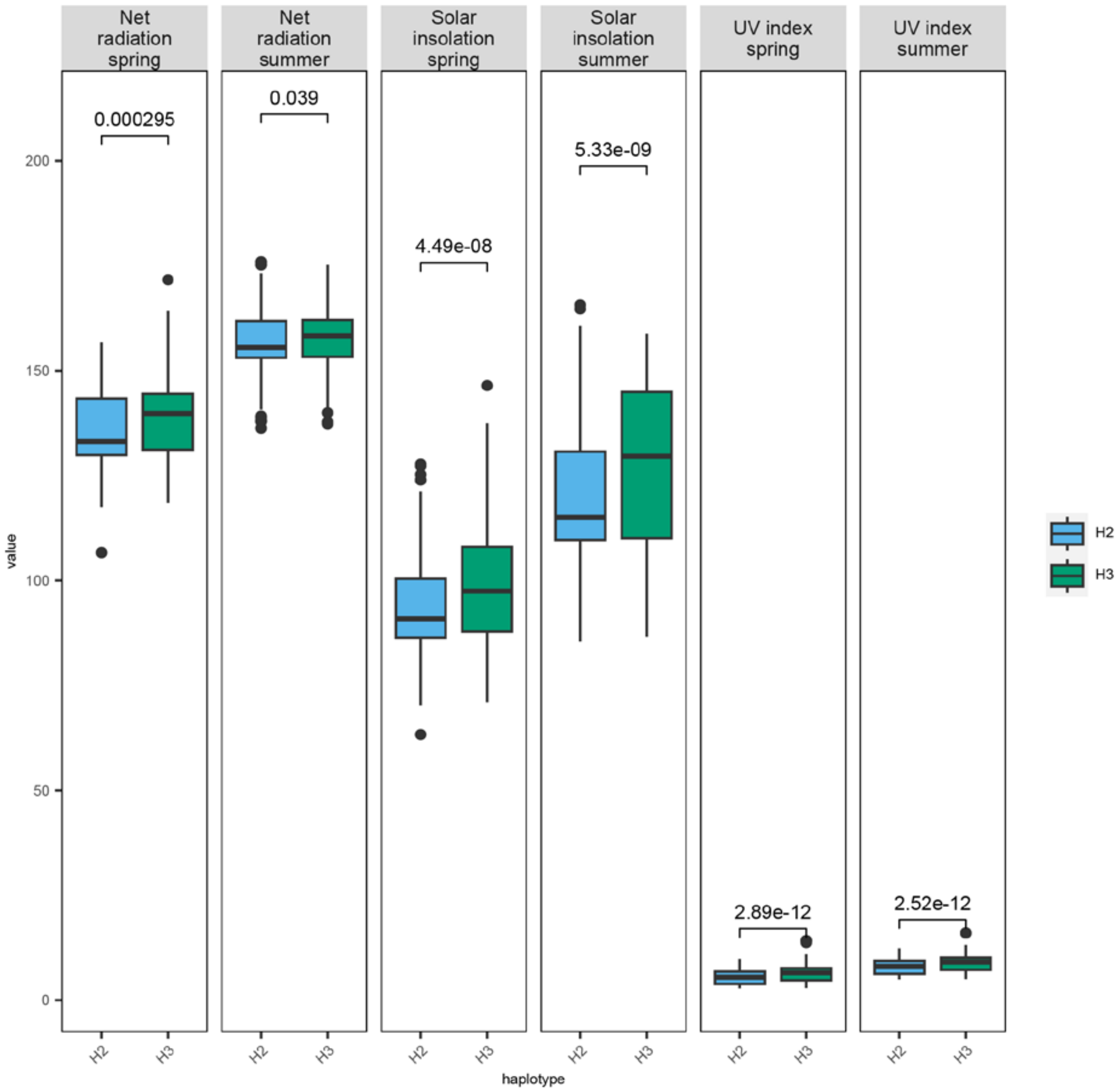

Supplemental Figure S2. Association of SNP haplotypes with local environment of Arabidopsis accessions. Boxplot showing the levels of UV index, solar insolation and net radiation in summer and spring relative to the local environment of Arabidopsis accessions with haplotypes H2 and H3 described in Figure 1D. Haplotype H2, which is associated with higher ascorbic acid levels, is more frequent in local environments with lower insolation than haplotype H3. Statistical significance was estimated with pairwise-wilcox tests and p-values are shown above the bracket. Boxplots show the median as the center line and the 1st and 3rd quartile as box limits. Whiskers extend as lines outwards from the box until the furthest data point, however with a maximum length of 1.5x the interquartile range (IQR). Points beyond 1.5x the IQR are displayed as individual outlier dots.

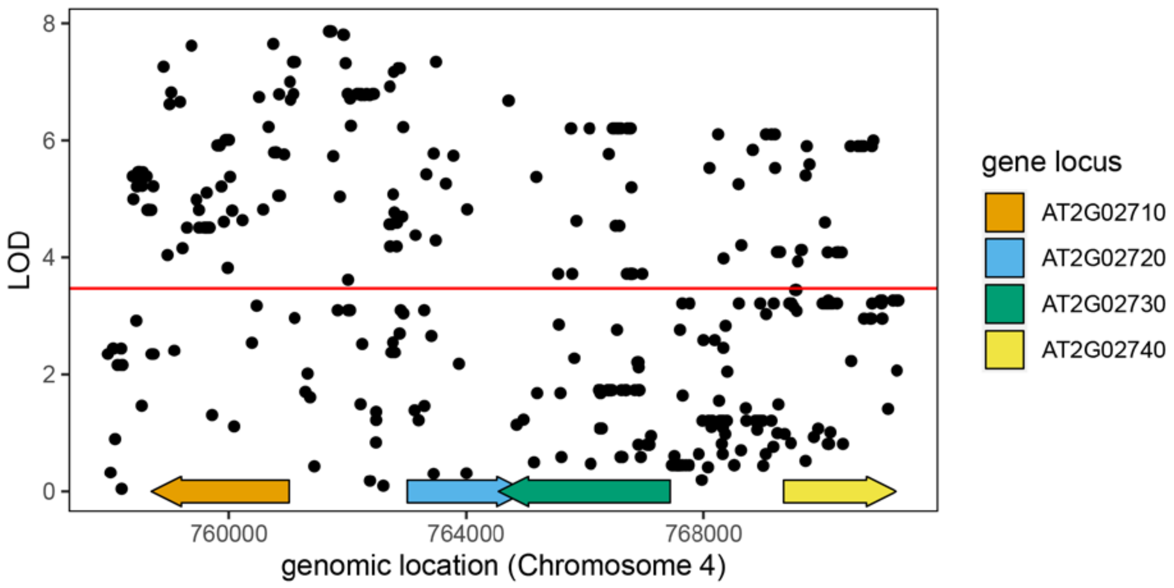

Supplemental Figure S3. Position of the SNPs identified in GWAS for total Asc under high light. Most of the leading SNPs lie upstream of PLP (AT2G02710), which is very close to AT2G02720 (encoding a pectate lyase family protein). Two other genes close to *PLP* are AT2G02730 (GRIP/coiled-coil protein, putative (DUF1664), and AT2G02740 (*WHIRLY 3_WHY3; PLASTID TRANSCRIPTIONALLY ACTIVE11_PTAC11*). Abbreviation: Logarithm of odds (LOD), "LOD score" refers to the log (base 10) of the odds of linkage.

```
Query  1    MSLTKSSESVFTEEEEEDSFSGRYTLWIKEALEELPHNFTITDPFISGHPIVFASLGFLK
60
            MSLTKSSESVFTEEEEEDSFSGRYTLWIKEALEELPHNFTITDPFISGHPIVFASLGFLK
Sbjct  1    MSLTKSSESVFTEEEEEDSFSGRYTLWIKEALEELPHNFTITDPFISGHPIVFASLGFLK
60

Query  61   MTGYSREEVIGRNGKVFQGPKTNRRSIMEIREAIREERSVQVSLLNYRKSGSPFWMLFHM
120
            MTGYSREEVIGRNGKVFQGPKTNRRSIMEIREAIREERSVQVSLLNYRKSGSPFWMLFHM
Sbjct  61   MTGYSREEVIGRNGKVFQGPKTNRRSIMEIREAIREERSVQVSLLNYRKSGSPFWMLFHM
120

Query  121  CPVFGKDDGKVTNFVAVQVPISGREHHRKKLRNVGDLSSDTSPTFGSCRREVCFGNFVCQ
180
            CPVFGKDDGKVTNFVAVQVPISGREHHRKKLRNVGDLSSDTSPTFGSCRREVCFGNFVCQ
Sbjct  121  CPVFGKDDGKVTNFVAVQVPISGREHHRKKLRNVGDLSSDTSPTFGSCRREVCFGNFVCQ
180

Query  181  DRALPVECDDDEQGLEDWEQCEASESEKLKATEAINNVLSILVHYSELSGRLVCGKRYCL
240
            DRALPVECDDDEQGLEDWEQCEASESEKLKATEAINNVLSILVHYSELSGRLVCGKRYCL
Sbjct  181  DRALPVECDDDEQGLEDWEQCEASESEKLKATEAINNVLSILVHYSELSGRLVCGKRYCL
240

Query  241  RGVDCLSSSLVISLGRIKQSFVLTNPCLPDMPIIYASDAFLTLTGYKRQEVLGQNCRFLS
300
            RGVDCLSSSLVISLGRIKQSFVLTNPCLPDMPIIYASDAFLTLTGYKRQEVLGQNCRFLS
Sbjct  241  RGVDCLSSSLVISLGRIKQSFVLTNPCLPDMPIIYASDAFLTLTGYKRQEVLGQNCRFLS
300

Query  301  GVDTDSSVLYEMKECILKGQSCTVQILNYSNRKDKSSFWNLLHISPVRNASGKTAYFVGV
360
            GVDTDSSVLYEMKECILKGQSCTVQILNY  RKDKSSFWNLLHISPVRNASGKTAYFVGV
Sbjct  301  GVDTDSSVLYEMKECILKGQSCTVQILNY--RKDKSSFWNLLHISPVRNASGKTAYFVGV
358

Query  361  QVEASCRNTEIKELRPETRQLSVVGAVRVAVRSSLMVTC  399
            QVEASCRNTEIKELRPETRQLSVVGAVRVAVRSSLMVTC
Sbjct  359  QVEASCRNTEIKELRPETRQLSVVGAVRVAVRSSLMVTC  397
```

Supplemental Figure S4. Protein alignment of PLPB and PLPA. The sequence alignment was performed using NCBI-blastP. Query refers to PLPB, and sbjct refers to PLPA. PLPB contains two additional amino acids (Ser and Asn) at position 330.

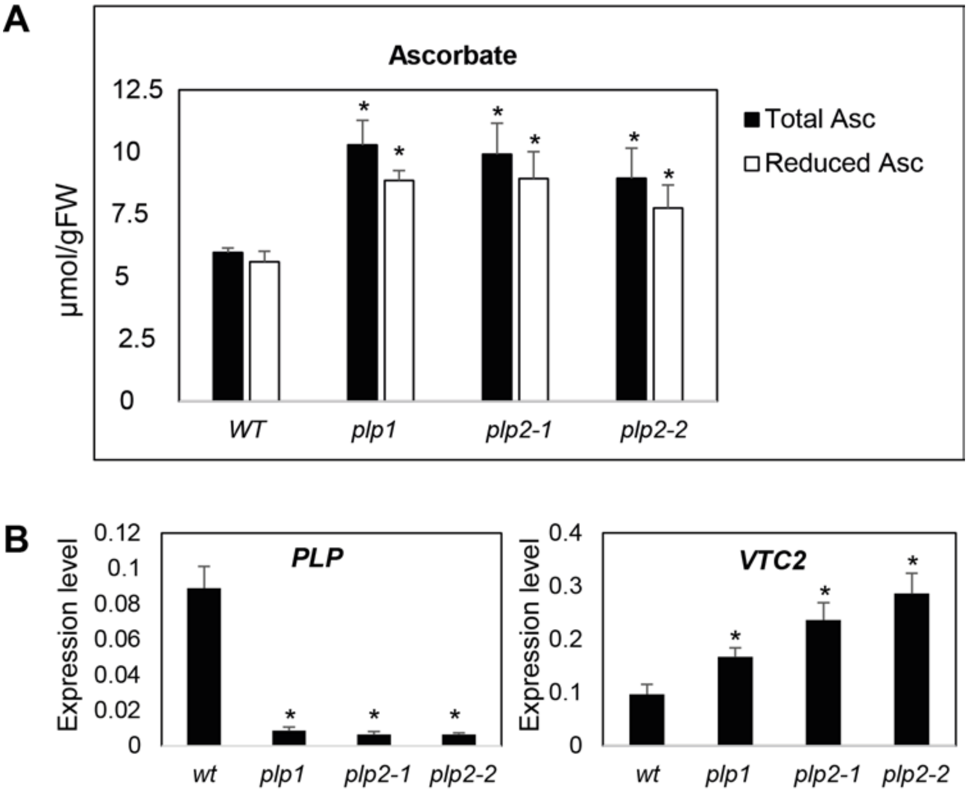

Supplemental Figure S5. Ascorbate and gene expression levels of the *plpb* knockouts grown on agar plates. (A) Rosettes (three weeks old) were harvested and used for ascorbate measurements. Error bars represent STDEV (n=4), and asterisks represent significant differences in each time point (P<0.05) calculated using Student'st-test. (B) Expression levels of *PLPB* and *VTC2* of the *plpb* mutants quantified by RT-qPCR normalized by $2^{-(CT\ gene - CT\ UBQ)}$. Error bars represent STDEV (n=6; 3 biological and 2 technical replicates), and asterisks represent significant differences in each time point (P<0.05) calculated using Student'st-test. Primers for the RT-qPCR are listed in Supplemental Table S2. Abbreviations: ascorbate (Asc), PAS/LOV PROTEIN (PLP), VITAMIN C DEFECTIVE2 (VTC2). Fresh weight (FW).

Ratio vs. WT	White LED			Red LED			Blue LED			Dark		
	Total Asc	Reduced Asc	DHA	Total Asc	Reduced Asc	DHA	Total Asc	Reduced Asc	DHA	Total Asc	Reduced Asc	DHA
plp1	1,47*	1,49*	1,32	1,37*	1,38*	1,30	1,31*	1,37*	0,88	1,39*	1,27*	3,10*
plp2-1	1,37*	1,39*	1,18	1,41*	1,42*	1,34	1,32*	1,39*	0,85	1,62*	1,47*	3,65*
plp2-2	1,49*	1,52*	1,24	1,39*	1,37*	1,49*	1,17*	1,19*	1,05	1,33*	1,26*	2,69*
cry1	1,07	1,10	0,82	1,04	1,02	1,28	0,79*	0,81*	0,66	0,94	0,81*	2,80*
cry2	1,23*	1,29*	0,86	1,24	1,25*	1,09	1,20*	1,23*	1,00	1,06	0,99	1,87
cry1cry2	1,16	1,17	1,08	1,01	0,98	1,25	0,81*	0,81*	0,78	0,88	0,83*	1,60

0,2 4

Supplemental Figure S6. Heatmap demonstrating the ascorbate levels of WT and the mutants exposed to different light qualities. Plants have been grown as described in Figure 8. Values demonstrate the fold changes of Total, reduced, and DHA levels of each mutant relative to the corresponding WT at each light condition. Asterisks represent significant differences between the mutants and the corresponding WT at each light condition, calculated using Student'st-test (P<0.05).

Supplemental Table S2. List of primers used in this study.

	Primer name	Primer sequence
T-DNA isolation	*plp1*_ left primer (LP)	TGTTTTTAATAAAATTTTGGTGAACG
	*plp1*_ right primer (LP)	CGGTGATTGTGAAATTGTGTG
	*plp2*_ left primer (LP)	TTTGTTTACGTGGAATCTCGG
	*plp2*_ right primer (LP)	TGAATTCGAAACACGAAATCC
	Left-border primer (LB)	GCGTGGACCGCTTGCTGCAACT
Cloning	pDONR207-VTC2-F	AAA AAAGCA GGCTCCACCATGTTGAAAATCAAAAGAGT TCCGA
	pDONR207-VTC2 C-R	CAAGAA AGCTGGGTCCTGAAGGACAAGGCACTC
	pDONR207-VTC5-F	AAAAAAGCAGGCTCCACCATGTTGTTGAAGATCAAAAGAGTT
	pDONR207-VTC5 C-R	CAAGAAAGCTGGGTCATTAGAGACAGCCTCTTCTTT
	pDONR207-PLP-F	AAAAAAGCAGGCTCCACCATGTCCTTAACGAAATCTTCAGA
	pDONR207-PLP C-R	CAAGAAAGCTGGGTCGCATGTCACCATCAATGAGC
	pDONR221 P2P3-PLP-F	GGGGACAACTTTGTATAATAAAGTTGGAATGTCCTTAACGAAATCTTCAGA
	pDONR221 P2P3-PLP-R	GGGGACCACTTTGTACAAGAAAGCTGGGT TTAGCATGTCACCATCAATGAG
	pDONR221 P1P4-VTC2-F	GGGGACA AGTTTGTACAAAAAAGCAGGCTTAATGTTGAAAATCAAAAGAGTTCCGA
	pDONR221 P1P4-VTC2-R	GGGGACAACTTTGTATAGAAAAGTTGGGTGCTGAAGGACAAGGCACTC
	pGADT7-PLP-F	GAGGCCAGTGAATTCATGTCCTTAACGAAATCTTCAGA
	pGADT7-PLP-R	CGATGCCCACCCGGGTTAGCATGTCACCATCAATGAG
RT-qPCR	*PLPB*-F	CCAATCCATGCTTACCAGACATGC
	PLPB-R	AACACTTCCTGTCTCTTGTAACCG
	VTC2-F	TCAGACTGCTGTGTTTGCCTTC
	VTC2-R	TGTTTCTCTGCGTAACACTGTGG
	UBQ10-F	GGCCTTGTATAATCCCTGATGAATAAG
	UBQ10-R	AAAGAGATAACAGGAACGGAAACATAGT
